# Somatic comorbidities in patients with schizophrenia

**DOI:** 10.1192/j.eurpsy.2024.1534

**Published:** 2024-08-27

**Authors:** N. Boussaid, M. Turki, M. Barkallah, A. Labyadh, N. Halouani, S. Ellouze, J. Aloulou

**Affiliations:** ^1^psychiatrie B; ^2^psychiatrie, CHU hedi Chaker, Sfax, Tunisia

## Abstract

**Introduction:**

Patients with schizophrenia have shown a high mortality rate, and life expectancy is shortened by 10-20 years. This seems to be mainly caused by metabolic and cardiovascular diseases. Several risk factors are identified, including sedentary lifestyle, poor diet, low socioeconomic status, cognitive dysfunction, and antipsychotics iatrogenicity.

**Objectives:**

We aimed to explore somatic pathologies reported in patients with schizophrenia, and to assess risk factors predisposing to these impairments.

**Methods:**

We conducted a retrospective descriptive and analytical study, based on clinical and psychiatric observations of 60 patients with schizophrenia, hospitalized in psychiatry “B” department, Hedi Chaker university hospital (Sfax, Tunisia), during the period between 2015 and 2017.

**Results:**

Among our patients, 38.3% suffered from somatic comorbidities: diabetes (21.7%), hypertension (15%), coronary disease (15%), hyperlipidaemia (15%), respiratory diseases (6.7%).

Tobacco consumption was reported in 53.3% of patients. It was significantly associated with the occurrence of cardiovascular diseases (p=0.036). Alcohol abuse was noted in 16.7%, while obesity was reported in 6.7% of patients.

Significant associations were found between obesity and diabetes (p=0.001), and between organic diseases and cognitive disorganisation (p=0.022). Somatic comorbidities were more frequent in patients with low socio-economic level (p=0.015).

Among our patients, 83.3% were treated with conventional antipsychotics while 38.3% were treated with atypical antipsychotics (AAP). We showed that AAP were associated with the occurrence of organic diseases (p=0.037).

**Conclusions:**

Physical health of patients with schizophrenia requires a serious attention. Coordinated care between psychiatrists and other healthcare professionals should monitor the physical health of these patients to prevent a premature death.

**Disclosure of Interest:**

None Declared

